# Study of Prescription Pattern of Antimicrobial Agents in the Medical Intensive Care Unit (MICU) of a Tertiary Care Hospital

**DOI:** 10.7759/cureus.107818

**Published:** 2026-04-27

**Authors:** Vishal Bonde, Pramod Shankpal, Samiksha M Lohi, Praneet R Sachdeo, Gaurav Yadav, Rosemarie D'souza

**Affiliations:** 1 Pharmacology, Government Medical College &amp; Hospital, Ambernath, IND; 2 Pharmacology and Therapeutics, Topiwala National Medical College and Bai Yamunabai Laxman Nair Charitable Hospital, Mumbai, IND; 3 Pharmacology, All India Institute of Medical Sciences, Nagpur, Nagpur, IND; 4 General Medicine, Topiwala National Medical College and Bai Yamunabai Laxman Nair Charitable Hospital, Mumbai, IND

**Keywords:** antibacterial stewardship program, antimicrobial resistance, critical care pharmacotherapy, drug utilization study, microbiological surveillance

## Abstract

Background

Medical intensive care units (MICUs) provide specialized care for critically ill patients requiring continuous monitoring. High staff density, frequent use of broad-spectrum antibiotics, and invasive procedures increase the risk of infections. Antimicrobial agents (AMAs), including antibacterial, antiviral, antifungal, and antiparasitic drugs, target specific pathogens. This study aimed to evaluate antimicrobial prescribing patterns in patients admitted to the MICU of a tertiary care hospital.

Methods

This prospective, single-center observational study was conducted over one year, with data collected from October 2023 to March 2024 in the MICU of a tertiary care hospital. A total of 238 MICU patients meeting the inclusion criteria were enrolled after obtaining informed consent. Data were collected from health management information system-generated prescriptions and patient interviews, including diagnosis, medical history, and treatment details. Demographic characteristics, duration of stay in MICU, comorbidities, and prescriptions were recorded and analyzed using descriptive statistics such as percentages, mean ± SD, and IQR.

Results

Among 238 patients, 55% were male, with a mean age of 38.47 ± 16.69 years. The majority (47.05%) belonged to the 21- to 40-year age group. Among 238 MICU patients, the most common cause of admission was acute febrile illness (13.87%), followed by lower respiratory tract infection (10.50%) and cardiogenic shock (7.98%). In our study, the most common duration of stay was 5 days (19.75%), followed by 3 days (18.49%) and 4 days (17.23%). Median duration of stay was 5 days (IQR: 3-7), with an overall range of 1 to 19 days. Among 1250 prescriptions, the total number of drugs prescribed was 10016, of which 2518 were AMAs. The average AMAs per prescription were 2.02 ± 1.01. The majority (36.40%) had at least two AMAs prescribed. Ceftriaxone (21.72%) and piperacillin-tazobactam (12.11%) were the most prescribed, with 67.71% used empirically and 23.07% for prophylaxis. Among 238 patients, 103 (43.28%) underwent blood culture testing. Of 113 cultures, 62.83% were positive and 37.17% negative. The most common isolates were *Streptococcus pneumoniae* (18, 25.35%), *Escherichia coli* (17, 23.94%), and *Klebsiella* spp. (13, 18.31%). *Streptococcus pneumoniae* (25.35%) showed the highest resistance to penicillin (88.89%), *Escherichia coli* (23.94%), and *Klebsiella* spp. (18.31%) to ceftriaxone (82.35% and 100%), while *Acinetobacter* spp. showed complete resistance to ceftriaxone. *Streptococcus pyogenes* showed the highest resistance to azithromycin and ceftriaxone (80% each). *Pseudomonas aeruginosa* showed multidrug resistance but remained sensitive to amikacin, imipenem, and colistin; MRSA isolates were sensitive to linezolid, tigecycline, and daptomycin.

Conclusion

The study revealed a tendency towards polypharmacy. Cephalosporins were most prescribed, followed by penicillin-beta-lactamase inhibitor combinations and glycopeptides. Most prescriptions were empirical, with fewer prophylactic and definitive therapies. The resistance pattern suggests overuse of broad-spectrum empirical therapy, limited culture-guided treatment, and possible gaps in infection control. Although some higher antibiotics remain effective, emerging resistance to carbapenems and colistin highlights the need for regular surveillance, rational prescribing, improved diagnostics, and strict antimicrobial stewardship.

## Introduction

The medical intensive care unit (MICU) manages critically ill patients requiring continuous monitoring; factors such as high patient turnover, frequent staff contact, invasive procedures, and broad-spectrum antibiotic use increase infection risk, resulting in a five to 10-fold higher incidence of nosocomial infections, with up to 30% developing hospital-acquired infections [1]. Antimicrobial agent (AMA) usage in the MICU is estimated to be about 10 times greater than in general hospital wards [2]. AMAs are classified into four types: antibacterial, antiviral, antifungal, and antiparasitic; each of them targets specific microorganisms, such as bacteria, viruses, fungi, or parasites, which helps physicians to select the most appropriate therapy for treating infections [3]. AMAs can be both synthetic and natural drugs that kill or halt the progression of microbes [4]. Nearly 5,000 AMAs have been discovered so far, but only about 100 are currently used in clinical practice to treat patients. In the MICU, patients usually receive multiple medications as part of their management, and AMAs are among the most commonly prescribed drugs [2]. However, most prescriptions of AMAs are empiric, based on physician knowledge and experience, which can lead to misuse of AMAs, increase AMA resistance, and increase the incidence of unnecessary side effects [3].

AMA resistance is an emerging threat and affects all individuals irrespective of age, sex, race, ethnicity, religion, or nationality. According to the World Health Organization and various authors' reports, most antimicrobial resistance is driven by improper, irrational, and widespread use of AMAts, with a likely increase in resistance to current therapies in the future [3, 5, 6]. Excessive or inappropriate use of AMAs contributes to rising treatment costs and antimicrobial resistance. In Europe, there has been a notable increase in extended-spectrum β-lactamase (ESBL)-producing bacteria, associated with the use of antibiotics such as ceftazidime, imipenem, and amoxicillin-clavulanic acid. Prolonged use of β-lactams and carbapenems has further led to the emergence of pandrug-resistant *Pseudomonas aeruginosa* and carbapenem-resistant Enterobacteriaceae [7]. Antimicrobial resistance threatens patients, healthcare systems, and the global economy, with multidrug-resistant bacteria increasingly common due to widespread antibiotic use, requiring coordinated efforts to reduce unnecessary use and prevent the spread of resistant pathogens [8].

The rise of antibiotic resistance has accelerated in recent decades due to bacterial adaptation via gene-mediated mechanisms that neutralize antibiotics [9], posing a major challenge with limited treatment options and driven largely by extensive antimicrobial use, particularly in intensive care units such as the MICU [10]. 

Despite extensive global data, region-specific evidence from critical care remains limited; this study evaluates antimicrobial use and resistance patterns in a tertiary care MICU in India to identify irrational use and assess the burden and trends of antimicrobial resistance.

The primary objective of this study was to evaluate the prescription pattern of AMAs in the MICU of the Department of Medicine at Topiwala National Medical College and Bai Yamunabai Laxman Nair Charitable Hospital, a tertiary care hospital in Mumbai, India, while the secondary objective was to evaluate the resistance pattern of AMAs used in the MICU.

## Materials and methods

This prospective, single-center, observational study was conducted over a period of one year and three months, from August 2023 to November 2024. The study was carried out in the MICU of the Department of Medicine at Topiwala National Medical College and Bai Yamunabai Laxman Nair Charitable Hospital, a government, urban, tertiary care teaching hospital in Mumbai, following approval from the Institutional Ethics Committee for Academic Research Projects (ECARP; Ref. No. ECARP/2023/150). The number of patients admitted to the MICU was approximately 26 patients per week. With a confidence level of 95% and a confidence interval (CI) of 5%, the sample size calculated for a six-month duration came to be 238 [11].

Adult patients (≥18 years) of either sex admitted to the MICU and receiving at least one AMA were included, provided their MICU stay was ≥24 hours. Patients unwilling or unable to provide informed consent were excluded. A convenience sampling method was used. Data were obtained from the health management information system-generated prescriptions (including diagnosis, history, and antimicrobial details) and patient/legally authorized representative (LAR) interviews using a predesigned, validated data collection form. Information on demographics, comorbidities, and antimicrobial therapy (drug, class, dose, frequency) was recorded by the co-investigator after obtaining written informed consent. Patients with samples sent for culture and sensitivity testing were prospectively followed until discharge, transfer, or death for microbiological analysis.

Statistical analysis was performed using Microsoft Excel 2019 (Microsoft Corp., Redmond, WA, USA). Categorical variables were expressed as frequencies and percentages. Antimicrobial resistance was defined as the proportion of isolates reported as resistant and expressed as percentage resistance for each agent, based on blood culture and sensitivity reports, to evaluate resistance patterns among commonly prescribed antimicrobials in the MICU.

## Results

Prescriptions were collected from 238 patients admitted to the MICU who had at least one AMA prescribed and were scrutinized for demographic profiles and drug prescription patterns over a period of six months, from October 2023 to March 2024. The results are presented below.

Demographic profile of the patients

Among 238 patients, 132 (55%) were male, and 106 (45%) were female, with a male-to-female ratio of 1.22:1. The mean age was 38.47 ± 16.69 years (range: 18-82 years). The most common age group was 21-40 years (n=112, 47.05%), followed by 41-60 years (n=70, 29.41%) (Table 1).

**Table 1:** Demographic details of the patients The data have been represented as N and %.

Variable	Category	Number of patients	Percentage (%)
Gender	Male	132	55.00
	Female	106	45.00
Age group	< 20 years	22	9.24
	21–40 years	112	47.05
	41–60 years	70	29.41
	61–80 years	30	12.60
	> 80 years	4	1.68

Clinical details of the patients

Among 238 MICU patients, acute febrile illness was the most common diagnosis (33, 13.87%), followed by lower respiratory tract infection (25, 10.50%), cardiogenic shock (19, 7.98%), ischemic heart disease (17, 7.14%), and diabetes mellitus (13, 5.46%); sepsis and dengue fever accounted for 11 cases each (4.62%), followed by congestive heart failure (nine, 3.78%), malaria (eight, 3.36%), upper respiratory tract infection (seven, 2.94%), left ventricular failure (six, 2.52%), acute gastroenteritis and acute respiratory failure (five each, 2.10%), encephalopathy and meningitis (four each, 1.68%), while other conditions constituted 61 cases (25.63%) (Table 2).

**Table 2:** Diagnosis of patients The data have been represented as N and %.

Diagnosis	Number of patients	Percentage (%)
Acute febrile illness	33	13.87
Lower respiratory tract infection	25	10.50
Cardiogenic shock	19	7.98
Ischemic heart disease	17	7.14
Diabetes mellitus	13	5.46
Sepsis	11	4.62
Dengue fever	11	4.62
Congestive heart failure	9	3.78
Malaria	8	3.36
Upper respiratory tract infection	7	2.94
Left ventricular failure	6	2.52
Acute gastroenteritis	5	2.10
Acute respiratory failure	5	2.10
Encephalopathy	4	1.68
Meningitis	4	1.68
Other	61	25.63

Out of 238 patients, 128 (53.78%) had no comorbidities. Of the 238 patients admitted to the MICU, 74 patients (31.09%) had one comorbidity, 32 patients (13.45%) had two comorbidities, two patients (0.84%) had three comorbidities, and two patients (0.84%) had four comorbidities. Overall, 110 out of 238 patients (46.22%) had comorbidities. Diabetes mellitus was the most common comorbidity, seen in 60 patients (25.21%), followed by hypertension, seen in 44 patients (18.49%), and ischemic heart disease, seen in 20 patients (8.4%) (Table 3).

**Table 3:** Frequency and distribution of comorbid conditions in the study population The data have been represented as N and %.

Variable	Category	Number of patients	Percentage (%)
Number of comorbidities	None	128	53.78
	One	74	31.09
	Two	32	13.45
	Three	2	0.84
	Four	2	0.84
Type of comorbidities	Diabetes mellitus	60	25.21
	Hypertension	44	18.49
	Ischemic heart disease	20	8.40
	Chronic obstructive pulmonary disease	12	5.04
	Rheumatic heart disease	10	4.20
	Anemia	10	4.20
	Epilepsy	6	2.52
	Bronchial asthma	6	2.52
	Rheumatoid arthritis	6	2.52
	Sjögren syndrome	2	0.84

**Figure 1:**
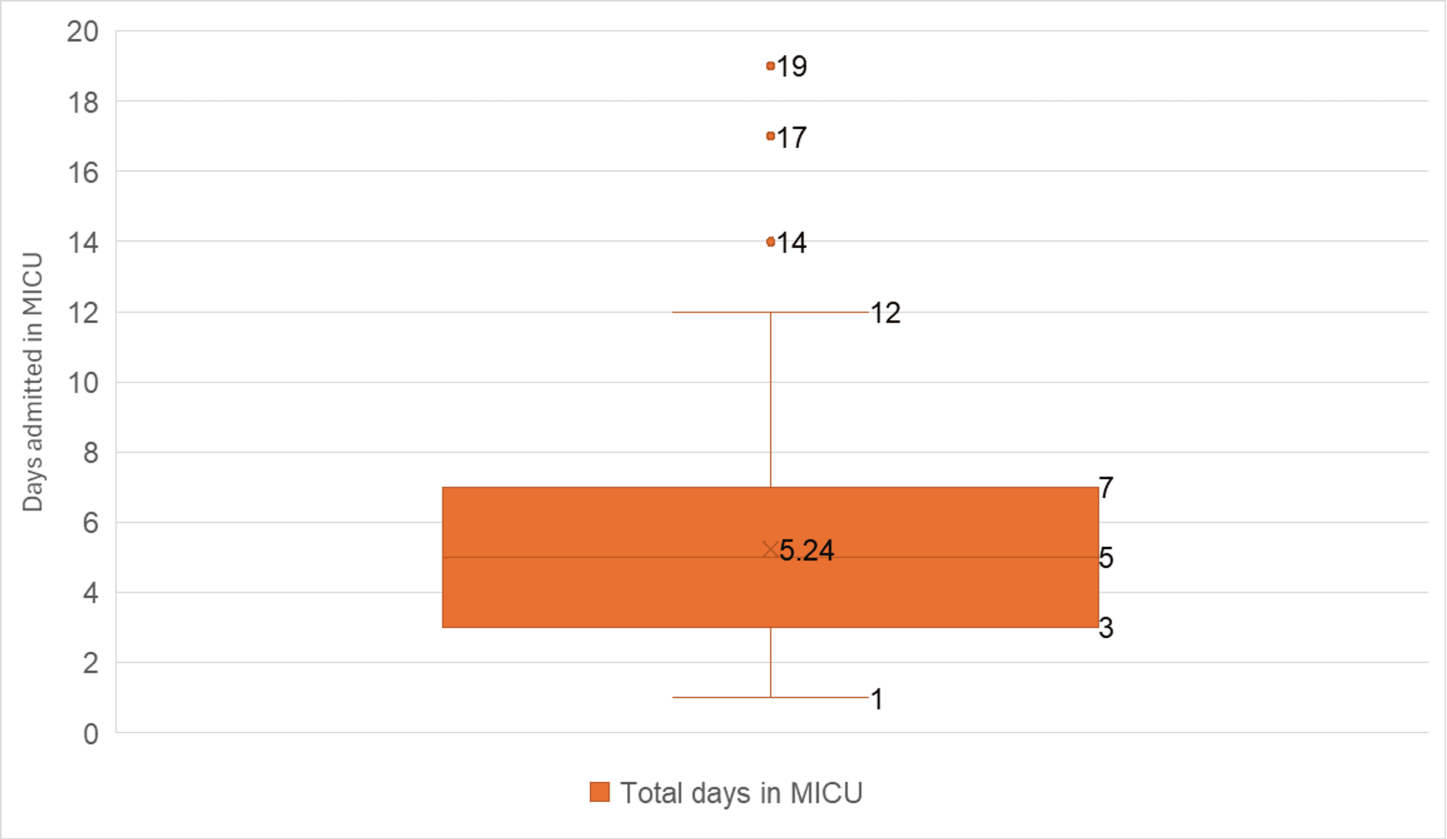
Duration of stay in the medical intensive care unit (MICUs) among the study population The data have been represented as N.

The mean MICU stay was 8.53 ± 5.37 days, with a median of five days (IQR: three to seven days); the most common durations were five days (47, 19.75%), three days (44, 18.49%), and four days (41, 17.23%), with an overall range of one to 19 days (Figure 1). 

Analysis of the prescription pattern

Pattern of the Number of AMAs Prescribed

A total of 238 MICU patients and 1,250 prescriptions (2,518 antimicrobials) were analysed, with a mean of 2.02 ± 1.01 agents per patient. Two antimicrobials were the most common, both at the prescription level (455, 36.4%) and patient level (86, 36.13%), followed by one (433, 34.64%; 66, 27.73%) and three agents (264, 21.12%; 58, 24.37%). Use of ≥4 agents was uncommon, and no patient received zero antimicrobials (Table 4).

**Table 4:** Total number of antimicrobial agents per prescription The data have been represented as N and %.

Total number of antimicrobial agents	Antimicrobial agents per prescription		Antimicrobial agents per patient	
	Number of prescriptions	Percentage (%)	Number. of patients	Percentage (%)
0	10	0.8	0	0
1	433	34.64	66	27.73
2	455	36.4	86	36.13
3	264	21.12	58	24.37
4	67	5.36	16	6.72
5	15	1.2	8	3.36
6	6	0.48	2	0.84
8	0	0	2	0.84
Total	1250	100	238	100

Frequency of Administration of AMAs

A total of 2,518 antimicrobials were prescribed. Ceftriaxone was the most common (N=547, 21.72%), followed by piperacillin-tazobactam (N=305, 12.11%), vancomycin (N=227, 9.02%), meropenem (N=209, 8.30%), and azithromycin (N=205, 8.14%). Other commonly used agents included artesunate (N=184, 7.31%), metronidazole (N=151, 6.00%), amoxicillin-clavulanic acid (injection: N=130, 5.16%; tablet: N=26, 1.03%), oseltamivir (N=117, 4.65%), and acyclovir (N=98, 3.89%), while remaining agents were used less frequently (Table 5).

**Table 5:** Frequency of administration of various antimicrobial agents AKT: anti-Koch's treatment The data have been represented as N and %.

Class of antimicrobial agents	Drug	Number of drugs prescribed	Percentage (%)
Cephalosporins	Ceftriaxone	547	21.72
Penicillins and β-lactamase inhibitors	Piperacillin + Tazobactam	305	12.11
	Amoxicillin + Clavulanic Acid (Injection)	130	5.16
	Amoxicillin + Clavulanic Acid (Tablet)	26	1.03
Glycopeptides	Vancomycin	227	9.02
	Teicoplanin	54	2.14
Carbapenems	Meropenem	209	8.30
Macrolides	Azithromycin	205	8.14
	Clarithromycin	38	1.51
Antimalarials	Artesunate	184	7.31
Nitroimidazoles	Metronidazole	151	6.00
Neuraminidase inhibitors	Oseltamivir	117	4.65
Antivirals	Acyclovir	98	3.89
Lincosamides	Clindamycin	75	2.98
Antimalarials (8-aminoquinoline)	Primaquine	48	1.91
Tetracyclines	Doxycycline	43	1.71
Rifamycin	Rifaximin	24	0.95
Fluoroquinolones	Ciprofloxacin	12	0.48
Antitubercular therapy	AKT	8	0.32
Penicillin	Penicillin	8	0.32
Glycylcycline	Tigecycline	6	0.24
Oxazolidinones	Linezolid	3	0.12
Total		2518	100

Frequency of Various Routes of Administration of AMAs

Out of 2,518 AMAs prescribed, 1,985 drugs (78.83%) were administered through the intravenous route, whereas 533 drugs (21.17%) were administered through the oral route.

Frequency of Drugs Prescribed as Monotherapy and Combination Therapy

A total of 2,518 AMAs were prescribed. The majority were used as monotherapy, accounting for 2,049 drugs (81.37%). Among combination therapies, piperacillin-tazobactam injection, a penicillin with a β-lactamase inhibitor, was the most common, with 305 prescriptions (12.11%), followed by amoxicillin-clavulanic acid given as injection in 130 cases (5.16%) and as tablet in 26 (1.03%). Antitubercular therapy, used as a fixed drug combination, accounted for eight prescriptions (0.32%) (Table 6).

**Table 6:** Frequency of administration of antimicrobial agents as monotherapy or combination therapy AKT: anti-Koch's treatment The data have been represented as N and %.

Antimicrobial agents in combination	Number of drugs	Percentage
Monotherapy	2049	81.37
Injection Piperacillin + Tazobactam	305	12.11
Injection Amoxicillin + Clavulanic Acid	130	5.16
Tablet Amoxicillin + Clavulanic Acid	26	1.03
AKT	8	0.32
Total	2518	100

Frequency of Administration of AMAs According to Indication, Categorized as Prophylactic, Empirical, and Definitive Therapy

Of the 2,518 AMAs prescribed, the majority were administered as monotherapy (2,049, 81.37%). Among individual agents, injectable piperacillin-tazobactam accounted for 305 prescriptions (12.11%), followed by injectable amoxicillin-clavulanic acid in 130 prescriptions (5.16%) (Table 7).

**Table 7:** Frequency of administration of antimicrobial agents according to indication, categorized as prophylactic, empirical, and definitive therapy The data have been represented as N and %.

Indication for antimicrobial agents	Number of prescribed antimicrobial agents	Percentage
Empirical	1705	67.71
Prophylactic	581	23.07
Definitive	232	9.21
Total	2518	100

Frequency of Resistance Pattern of Various AMAs

**Figure 2:**
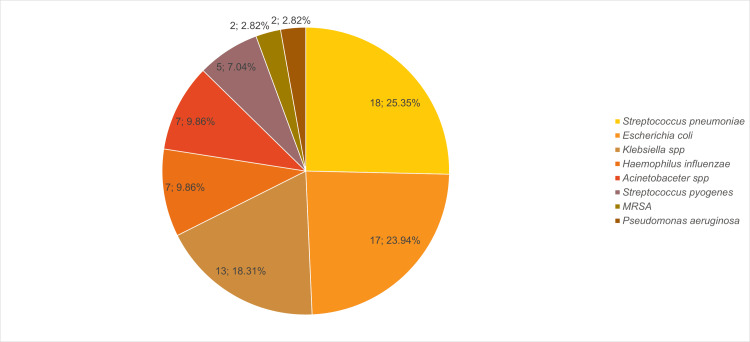
Frequency of organisms isolated from blood culture sensitivity samples MRSA: methicillin-resistant *Staphylococcus aureus* The data have been represented as N and %.

Among the 238 patients included in the study, 103 (43.28%) underwent blood culture and sensitivity testing. A total of 113 blood culture samples were analysed, of which 42 (37.17%) showed no growth, while 71 (62.83%) were culture positive. Among the positive isolates (n = 71), *Streptococcus pneumoniae* was the most frequently identified organism, accounting for 18 cases (25.35%), followed by* Escherichia coli* in 17 cases (23.94%) and *Klebsiella *spp. in 13 cases (18.31%) (Figure 2).

**Figure 3:**
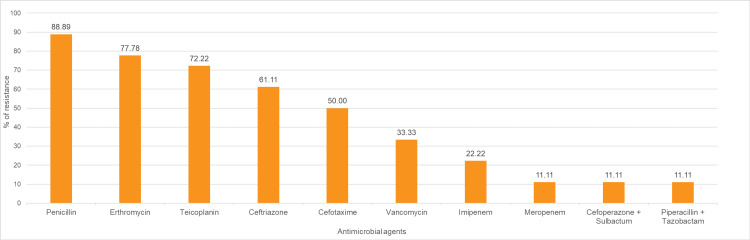
Antibiotic resistance pattern of Streptococcus pneumoniae The data have been represented as %.

The pattern of antimicrobial resistance in *Streptococcus pneumoniae,* which were isolated from 18 (25.35%) blood culture samples out of 71 blood culture samples, with a maximum of which are resistant to penicillin (n=16; 88.89%), followed by erythromycin (n=14; 77.78%), followed by teicoplanin (n=13; 72.22%), followed by ceftriaxone (n=11; 61.11%). *Streptococcus pneumoniae,* which were isolated from blood culture samples, were least resistant to cefoperazone + sulbactam, piperacillin + tazobactam combination and meropenem (n=2; 11.11% each), followed by Imipenem (n=4; 22.22%), followed by vancomycin (n=6; 33.33%) and cefotaxime (n=9; 50%) (Figure 3). 

**Figure 4:**
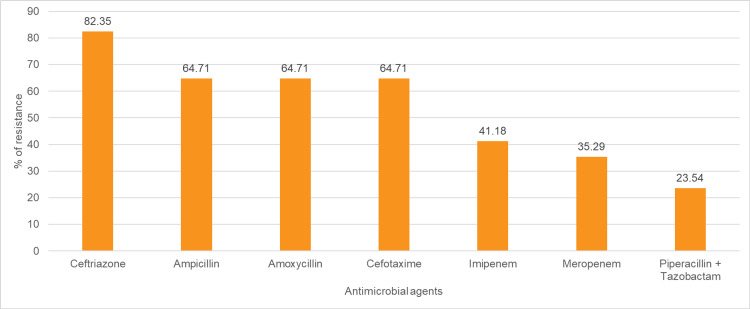
Antibiotic resistance pattern of Escherichia coli The data have been represented as %.

The pattern of antimicrobial resistance in *Escherichia coli,* which were isolated from 17 (23.94%) blood culture samples out of 71 blood culture samples, with the highest resistance to ceftriaxone (n=14; 82.35%), followed by penicillin (n=11; 64.71%), followed by cefotaxime (n=11; 64.71%). *Escherichia coli* were least resistant to piperacillin + tazobactam (n=4; 23.53%), followed by meropenem (n=6; 35.29%) and imipenem (n=7; 41.18%) (Figure 4).

**Figure 5:**
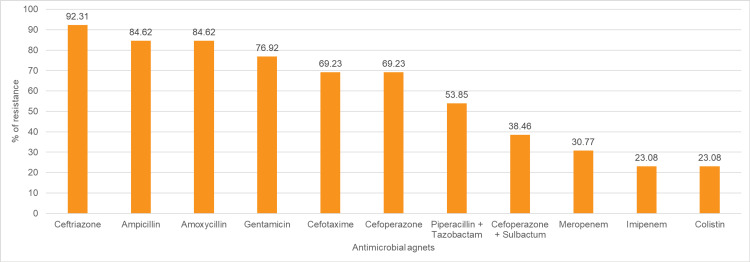
Antibiotic resistance pattern of Klebsiella spp. The data have been represented as %.

*Klebsiella *spp., which were isolated in 13 (18.31%) out of 71 blood culture samples, were highly resistant to ceftriaxone (n=12; 92.31%), followed by penicillin (n=11; 84.62%), followed by gentamicin (n=10; 76.92%), and cefotaxime and cefoperazone (n=9; 69.23%). *Klebsiella *spp. were least resistant to colistin and imipenem (n=3; 23.08% each), followed by meropenem (n=4; 30.77%), followed by cefoperazone + sulbactam (n=5; 38.46%), and piperacillin + tazobactam, amikacin (n=7; 53.85%) (Figure 5).

High antimicrobial resistance was observed in *Acinetobacter *spp., with complete resistance to ceftriaxone (N=7, 100%) and high resistance to amikacin and aztreonam (N=6, 85.71% each); lower resistance was noted with minocycline and imipenem (N=3, 42.86% each) and meropenem (N=4, 57.14%). *Streptococcus pyogenes* showed the highest resistance to azithromycin and ceftriaxone (N=4, 80% each), followed by cefotaxime (60%), while lower resistance (N=1, 20% each) was seen with linezolid, meropenem, imipenem, and piperacillin-tazobactam. Both *Pseudomonas aeruginosa* isolates were resistant to ceftriaxone, cefoperazone, ceftazidime, amoxicillin-clavulanate, gentamicin, meropenem, and piperacillin-tazobactam, respectively, but sensitive to amikacin, imipenem, and colistin. Methicillin-resistant *Staphylococcus aureus* (MRSA) isolates (N=2) were sensitive to linezolid, tigecycline, and daptomycin, respectively, with intermediate susceptibility to colistin, vancomycin, and teicoplanin, respectively.

## Discussion

Among 238 MICU patients, 55% (n=132) were male and 45% (n=106) were female (M:F = 1.22:1), showing male predominance, consistent with studies by Rajathilagam et al. (60% males, 40% females), Anand et al. (66.6% males, 33.4% females), Bhatia et al. (55.77% males, 44.23% females), and Mondal et al. (M:F = 1.45), while Saxena et al. reported female predominance [2, 6, 12, 13].

The age of patients ranged from 18 to 82 years, with a mean of 38.47 ± 16.69 years. In the study conducted by Mondal et al., the mean age was 63.32 ± 17.93 years, comparable to Saxena et al. (mean 39.5 years; 20-39 years: 48%), whereas Williams et al. reported 49 ± 19.5 years with 46-60 years (33%) predominating, in contrast to our study, where 21-40 years (n=112, 47.05%) was the most common age group [6, 10, 14]. This tertiary care MICU study, receiving referrals from primary and secondary centers, showed a relatively younger population (mean age 38.47 ± 16.69 years), possibly reflecting disease epidemiology and factors such as lifestyle, genetics, and urban living, which may influence disease progression and outcomes.

We studied the disease distribution of the study population and divided them based on diagnosis. Out of 238 patients, 33 (13.87%) patients were admitted to the MICU for acute febrile illness, followed by lower respiratory tract infection (n=25, 10.50%), followed by cardiogenic shock (n=19, 7.98%), followed by ischemic heart disease (7.14%) (Table 2). The result of our study is in contrast to the study conducted by Tran et al., in which they found the most common primary diagnosis for admission to MICU was respiratory failure (57.7%), followed by hypertension (36.5%) and diabetes mellitus (25.9%) [15]. The observed variation in diagnosis prevalence compared to existing literature may be attributed to differences in population demographics, healthcare access, disease burden, and referral patterns in a tertiary care setting in a developing country.

In our study, 46.22% (110/238) patients had comorbidities, with 31.09% (74) having one and 13.45% (32) having two, which is comparable to the study by Anand et al. (48.9%, 32.8%, and 12.3%, respectively) [2]; diabetes mellitus (60, 25.21%) was the most common, followed by hypertension (44, 18.49%) and ischemic heart disease (20, 8.40%) (Table 3). The study conducted by Anand et al. found that hypertension was the most common comorbidity, followed by diabetes mellitus and ischemic heart disease, which slightly contradicts our finding [2]. The study conducted by Rajathilagam et al. found the most common comorbidity was diabetes mellitus, followed by hypertension and coronary artery disease [12]. The high coexistence of diabetes and hypertension in Indian adults is driven by shared metabolic risk factors (insulin resistance and inflammation) along with aging, urbanization, sedentary lifestyle, and dietary and socioeconomic transitions, with higher prevalence in older, urban, and higher-income populations, underscoring the need for integrated public health strategies.

The mean MICU stay was 8.53 ± 5.37 days with a median of five days (IQR: three to seven days), most commonly five days (19.75%), followed by three (18.49%) and four days (17.23%), with an overall range of one to 19 days. This is in accordance with the study conducted by Williams et al., in which 57.5% of patients had one to five days of ICU stay [14]. The study conducted by Rajathilagam et al. found that the average stay of patients in the ICU was 5.5 days ± 2.4 days (mean ± SD) [12]. The wide range reflects a significant variation in prescribing practices, which could be attributed to differences in disease severity and the presence of comorbidities, underscoring the need for more standardized antimicrobial stewardship protocols.

The average number of AMAs prescribed in our study was 2.02 ± 1.01 (mean ± SD). The studies conducted by Marasine et al. and Anand et al. found the average number of antibiotics prescribed to patients was 1.73 and 2.32 ± 0.99 (mean ± SD), respectively [1, 2]. This discrepancy may be due to differences in study settings, patient populations, prescribing practices, and illness severity. Of 1,250 prescriptions analyzed, two AMAs were the most common (455, 36.40%), followed by one agent (433, 34.64%) and three agents (264, 21.12%). These findings differ from those reported by Yadav et al., where three, two, one, and four or five antibiotics were prescribed in 43%, 27%, 18%, and 4% of patients, respectively, and by Anand et al., where one, two, and three agents were used in 33.9%, 27.1%, and 11.9% of patients, respectively [2, 16]. The higher antimicrobial use may reflect the tertiary care setting with more severe infections, along with differences in patient demographics, infection types, resistance patterns, institutional protocols, diagnostic support, and evolving treatment guidelines.

In our study, the most commonly prescribed class of AMAs was cephalosporins (n=547, 21.72%), followed by penicillin with β-lactamase inhibitors (n=461, 18.31%), followed by glycopeptides (n=276, 11.16%) (Table 5). This is in accordance with the study conducted by Mondal et al., where they found that the most common classes of AMAs prescribed to patients who were admitted to MICU were penicillin (51.87%) and cephalosporins (45.78%) [6]. These findings likely reflect the widespread use, broad-spectrum activity, availability, and perceived effectiveness of these agents in critically ill patients, with frequent use of cephalosporins and β-lactam/β-lactamase inhibitor combinations indicating empirical therapy targeting both Gram-positive and Gram-negative organisms.

Among 2,518 AMAs, ceftriaxone was most commonly prescribed (21.72%), followed by piperacillin-tazobactam (12.11%), vancomycin (9.02%), meropenem (8.30%), azithromycin (8.14%), artesunate (7.31%), and metronidazole (6%) (Table 5). These findings are broadly consistent with previous studies, though variations exist. Marasine et al. reported piperacillin-tazobactam (n=71) as the most common, followed by ceftriaxone (n=54), metronidazole (n=45), and doxycycline (n=38), while Mondal et al. reported piperacillin (37.03%), ceftriaxone (33.28%), and levofloxacin (22.5%) [1, 6]. Similarly, Anand et al. reported ceftriaxone, piperacillin-tazobactam, metronidazole, linezolid, and amoxicillin-clavulanic acid as the most common, while Williams et al. and Bhatia et al. also observed variations with piperacillin-tazobactam and meropenem among the frequently used agents [2, 13, 14]. Differences in prescribing patterns may reflect variations in patient profiles, institutional practices, local resistance patterns, and clinician preferences, with frequent ceftriaxone use likely due to its broad-spectrum activity and affordability, while increased use of vancomycin and meropenem may indicate early escalation or a higher burden of resistant infections.

In the present study, among the 2,518 AMAs prescribed, the majority (n = 1,985, 78.83%) were administered via the intravenous route, whereas 533 agents (21.17%) were administered orally. These findings are consistent with those reported by Anand et al., where 77% of AMAs were administered parenterally and 23% orally [2]. This pattern indicates that IV use predominates in MICU settings; however, a timely IV-to-oral switch is essential for antimicrobial stewardship to reduce complications and costs and improve outcomes.

In the present study, out of a total of 2,518 AMAs prescribed, 2,076 (82.45%) were administered as monotherapy, while 442 (17.55%) were prescribed as part of combination therapy regimens (Table 6). Among fixed-dose combinations, injection piperacillin + tazobactam was most commonly prescribed (305, 12.11%), followed by injection amoxicillin + clavulanic acid (130, 5.16%) (Table 6). The predominance of piperacillin + tazobactam in our study aligns with earlier research, with Mondal et al. reporting it as the most frequently prescribed a fixed drug combination (37.03%), followed by ceftriaxone + sulbactam, highlighting a similar preference for broad-spectrum β-lactam/β-lactamase inhibitor combinations, while Rajathilagam et al. also observed frequent use of amoxicillin + sulbactam and cefoperazone + sulbactam, followed by anti-tuberculosis drug combinations [6, 12]. These findings indicate regional variation but underscore the common reliance on β-lactam/β-lactamase inhibitor combinations and the increasing use of combination therapy to enhance efficacy and overcome resistance.

In our study, of 2,519 antimicrobials, most were prescribed empirically (1,705, 67.71%), followed by prophylactic (581, 23.07%) and definitive therapy (233, 9.21%), consistent with Marasine et al. (72%, n=113; 18.5%, n=29; 9.6%, n=15), reflecting common reliance on empirical treatment in clinical practice [1]. Their study similarly reflects reliance on empirical therapy, likely due to the need for early treatment and limited or delayed microbiological diagnostics. These findings highlight the importance of strengthening diagnostic support and antimicrobial stewardship to optimize definitive therapy and reduce unnecessary broad-spectrum use and antimicrobial resistance.

In our study, blood culture and sensitivity testing were performed in 103 of 238 patients (43.28%; 113 samples), indicating that more than half were treated empirically without microbiological confirmation. Similar variability has been reported, with rates of 24% by Saxena et al. and 57.3% by Marasine et al., reflecting differences across settings [1, 10]. These variations highlight inconsistent use of microbiological investigations. Although empirical therapy is often necessary in acute settings, limited use of culture and sensitivity testing may reduce treatment precision and contribute to prolonged or inappropriate antimicrobial use.

Of 113 cultures, 71 (62.83%) were positive; *Streptococcus pneumoniae* (25.35%) was most common, followed by *Escherichia coli* (23.94%) and *Klebsiella *spp. (18.31%), with other isolates less frequent (Figure 2). These findings differ from other studies, where Marasine et al. reported *Escherichia coli *as the most common isolate (n=10), followed by *Pseudomonas *spp. (n=4) and *Acinetobacter *spp. (n=2), while Tran et al. found *Acinetobacter baumannii* (31%) as predominant, followed by *Klebsiella pneumoniae* (21.5%) and *Pseudomonas aeruginosa* (16.3%), with *Escherichia coli* and *Staphylococcus aureus* each at 5% [1, 15]. The variation in microbial profiles across these studies may be attributed to differences in geographic regions, local antimicrobial usage patterns, infection control practices, patient populations, and the prevalence of community- versus hospital-acquired infections. Such differences underscore the importance of ongoing local surveillance to guide empirical antibiotic therapy in critically ill patients.

Among 71 positive cultures, *Streptococcus pneumoniae* (25.35%) showed high resistance to penicillin (88.89%), erythromycin (77.78%), teicoplanin (72.22%), and ceftriaxone (61.11%); moderate resistance to cefotaxime (50%) and vancomycin (33.33%); and lower resistance to imipenem (22.22%), cefoperazone-sulbactam, and meropenem (11.11%), underscoring the need for culture-guided therapy (Figure 3). Direct comparison is limited, as Streptococcus pneumoniae was not reported in comparable studies, emphasizing the need for local surveillance and region-specific data to guide empirical therapy and optimize antibiotic use.

*Escherichia coli *(23.94%) showed the highest resistance to ceftriaxone (82.35%), followed by penicillin and cefotaxime (64.71% each), with lower resistance to piperacillin-tazobactam (23.53%), meropenem (35.29%), and imipenem (41.18%) (Figure 4). These findings are partially consistent with studies by Bhatia et al. and Saxena et al., where *Escherichia coli *showed high resistance to cephalosporins (60-89%), fluoroquinolones (85%), and other antibiotics, with comparatively lower resistance to carbapenems (8-40%) and colistin (11%), although higher resistance to piperacillin + tazobactam (23-70%) was noted in some settings [10, 13]. Variations in resistance patterns may be due to differences in prescribing practices, infection control, prior antibiotic exposure, and prevalence of multidrug-resistant strains, along with factors such as sample size, clinical setting, and comorbidities, highlighting the need for local surveillance and institution-specific antibiotic policies.

In the present study, *Klebsiella *spp. were isolated from 13 (18.31%) out of 71 blood culture samples and showed high resistance to ceftriaxone (100%), penicillin (84.62%), and gentamicin (76.92%). Resistance was lowest to colistin and imipenem (23.08%), followed by meropenem (30.77%), and moderate to piperacillin + tazobactam and amikacin (53.85%). Similar trends were observed across studies, including Bhatia et al., where *Klebsiella pneumoniae* showed 100% resistance to multiple antibiotics with lower resistance to colistin (22%) and tigecycline (10%); Tran et al., who reported high resistance to amikacin (94.9%), carbapenems (74-80%), and third-generation cephalosporins (73-83%) with lower resistance to cefoperazone-sulbactam (21%) and ertapenem (53.6%), and Saxena et al., where *Klebsiella *spp. demonstrated high resistance to cephalosporins (83%), ciprofloxacin and gentamicin (74%), piperacillin + tazobactam (70%), and moderate resistance to carbapenems and amikacin (61-66%) [10, 13, 15]. High resistance across studies may be attributed to injudicious use and misuse of broad-spectrum antibiotics, poor antimicrobial stewardship, and cross-transmission of resistant strains, especially in intensive care units.

In the present study, *Acinetobacter *spp. showed high antimicrobial resistance, with 100% resistance to ceftriaxone, 85.71% to amikacin and aztreonam, 71.43% to ampicillin + sulbactam, ceftazidime, gentamicin, and ciprofloxacin, while lower resistance was observed with minocycline and imipenem (42.86% each) and meropenem (57.14%). Comparable findings were reported by Tran et al. and Saxena et al., where *Acinetobacter *isolates showed high resistance to ceftriaxone (95.2%), ciprofloxacin (95.2%), ceftazidime (93.2% and 87.5%), imipenem (93.2% and 87.5%), meropenem (90.5% and 81%), piperacillin + tazobactam (95.0% and 75%), amikacin (77.8% and 75%), and gentamicin (84.1% and 75%) [10, 15]. These findings highlight the alarming multidrug resistance in *Acinetobacter *spp. to β-lactams, aminoglycosides, and fluoroquinolones, likely due to rapid acquisition of resistance genes via plasmids, integrins, and transposons, along with its ability to persist in hospital environments, leading to nosocomial infections and limited therapeutic options.

The observed resistance pattern of *Streptococcus pyogenes* in blood cultures, with high resistance to azithromycin and ceftriaxone (80%) and moderate resistance to cefotaxime (60%), can be attributed to several factors. Injudicious use and empirical prescribing, along with inappropriate dosing and duration, contribute to resistance, further compounded by limited antimicrobial stewardship. Lower resistance to linezolid, meropenem, imipenem, and piperacillin-tazobactam (20%) likely reflects their restricted use and better stewardship.

In our study, *Pseudomonas aeruginosa* was isolated from two out of 71 blood culture samples. The isolates were resistant to ceftriaxone, cefoperazone, ceftazidime, aztreonam, and minocycline. Intermediate susceptibility was observed with cefepime, ceftazidime + clavulanic acid, gentamicin, meropenem, and piperacillin + tazobactam, while they remained sensitive to amikacin, imipenem, and colistin. In comparison, studies by Tran et al. and Saxena et al. reported variable susceptibility patterns in Pseudomonas aeruginosa, with higher sensitivity to ceftriaxone (100%), meropenem (86.2% and 40%), imipenem (79.3% and 60%), gentamicin (80% and 20%), and ciprofloxacin (80% and 70%), while lower susceptibility was noted for piperacillin + tazobactam (32.1% and 30%), cefepime (61.9%), amikacin (20%), and colistin (3.4%) [10, 15]. These differences in susceptibility patterns may be due to variations in local antimicrobial use, infection control practices, and the prevalence of multidrug-resistant strains, with regional prescribing habits, genetic diversity, and selective pressure influencing resistance trends, highlighting the need for continuous local surveillance and antimicrobial stewardship.

In our study, two blood culture samples tested positive for MRSA. The isolates were sensitive only to linezolid, tigecycline, and daptomycin, with intermediate sensitivity to colistin, vancomycin, and teicoplanin. This limited susceptibility in MRSA reflects increasing treatment challenges due to antibiotic indiscriminate use, poor infection control, and emerging resistance, with intermediate glycopeptide sensitivity suggesting early resistance, underscoring the need for judicious antibiotic use, regular monitoring, and strict infection control.

Strengths of the study

This study represents a recent evaluation of antimicrobial prescribing patterns in the MICU of a tertiary care hospital at our institute, offering meaningful insight into contemporary drug utilization practices in a critical care setting. In addition, the assessment of antimicrobial resistance patterns based on blood culture isolates provides clinically relevant information that can inform empirical therapy and strengthen antimicrobial stewardship strategies. The inclusion of a heterogeneous patient population with varied comorbidities further enhances the external validity and applicability of the findings.

Limitations of the study

The study was conducted at a single tertiary care center, and the use of non-probability sampling with the inclusion of only available patients may have introduced selection bias, potentially limiting the representativeness and generalizability of the findings. Additionally, the analysis was restricted to prescription data and blood culture reports during MICU stay, without follow-up microbiological data after discharge, transfer, or death, which may have affected the comprehensive assessment of antimicrobial utilization and resistance patterns.

## Conclusions

This study highlights important antimicrobial prescribing patterns and resistance trends in a tertiary care MICU setting. Empirical antimicrobial use was predominant, with frequent use of cephalosporins, β-lactam/β-lactamase inhibitor combinations, and glycopeptides, often as multidrug regimens and administered via the intravenous route. A substantial burden of antimicrobial resistance was observed among common pathogens, particularly *Streptococcus pneumoniae, Escherichia coli, Klebsiella *spp., and *Acinetobacter *spp., with relatively lower resistance to selected reserve agents. These findings emphasize the need for early microbiological confirmation wherever feasible, rational selection of antimicrobials, and timely de-escalation based on culture and sensitivity results. Strengthening antimicrobial stewardship practices, ensuring appropriate use of broad-spectrum agents, and improving access to reliable microbiological diagnostics are critical to optimize patient outcomes and reduce the emergence of resistance in critical care settings.

Future studies should focus on evaluating the relationship between antimicrobial prescribing patterns and clinical outcomes, including duration of stay and mortality, to further support evidence-based antimicrobial use.
